# Persistent Expression of Serotonin Receptor 5b Alters Breathing Behavior in Male MeCP2 Knockout Mice

**DOI:** 10.3389/fnmol.2018.00028

**Published:** 2018-02-20

**Authors:** Steffen Vogelgesang, Marcus Niebert, Anne M. Bischoff, Swen Hülsmann, Till Manzke

**Affiliations:** ^1^DFG-Research Center Nanoscale Microscopy and Molecular Physiology of the Brain (CNMPB), University of Göttingen, Göttingen, Germany; ^2^Institute of Neuro- and Sensory Physiology, University of Göttingen, Göttingen, Germany; ^3^Clinic for Anesthesiology, University Medical Göttingen, Göttingen, Germany

**Keywords:** Rett syndrome, MeCP2, GPCR, 5-ht_5b_ receptor, cAMP regulation, breathing disturbances

## Abstract

Mutations in the transcription factor methyl-CpG-binding protein 2 (MeCP2) cause the neurodevelopmental disorder Rett syndrome (RTT). Besides many other neurological problems, RTT patients show irregular breathing with recurrent apneas or breath-holdings. MeCP2-deficient mice, which recapitulate this breathing phenotype, show a dysregulated, persistent expression of G-protein-coupled serotonin receptor 5-ht_5b_ (*Htr5b*) in the brainstem. To investigate whether the persistence of 5-ht_5b_ expression is contributing to the respiratory phenotype, we crossbred MeCP2-deficient mice with 5-ht_5b_-deficient mice to generate double knockout mice (*Mecp2^−/y^*;*Htr5b*^−/−^). To compare respiration between wild type (WT), *Mecp2^−/y^* and *Mecp2^−/y^*;*Htr5b*^−/−^ mice, we used unrestrained whole-body plethysmography. While the breathing of MeCP2-deficient male mice (*Mecp2^−/y^*) at postnatal day 40 is characterized by a slow breathing rate and the occurrence of prolonged respiratory pauses, we found that in MeCP2-deficient mice, which also lacked the 5-ht_5b_ receptor, the breathing rate and the number of pauses were indistinguishable from WT mice. To test for a potential mechanism, we also analyzed if the known coupling of 5-ht_5b_ receptors to G_i_ proteins is altering second messenger signaling. Tissue cAMP levels in the medulla of *Mecp2^−/y^* mice were decreased as compared to WT mice. In contrast, cAMP levels in *Mecp2^−/y^*;*Htr5b*^−/−^ mice were indistinguishable from WT mice. Taken together, our data points towards a role of 5-ht_5b_ receptors within the complex breathing phenotype of MeCP2-deficient mice.

## Introduction

The neurodevelopmental Rett syndrome (RTT) occurs primarily in females with an incidence of 1:10,000 live births and presents as a delayed regression after 6–18 months of apparently normal development (Rett, 1966; Julu et al., 2001). The disturbances typically start with early autonomic dysfunctions, including breathing abnormalities that are considered a potential cause of sudden death (Kerr et al., 1997). The progressed phenotype manifests with neurological symptoms such as stereotypic hand movements, seizures and mental retardation with loss of language skills. RTT is caused by nonsense, missense or frameshift mutations, as well as large deletions of the human X-chromosomal gene methyl-CpG-binding protein 2 (*MECP2*; Amir et al., 1999; Philippe et al., 2006), which encodes the transcription factor MeCP2. MeCP2 acts in a DNA-methylation-dependent manner by repressing or activating gene transcription (Lewis et al., 1992; Nan et al., 1997; Chahrour et al., 2008). MeCP2 is considered to be important during brain development, it accumulates most abundantly in post-mitotic adult neurons, where it is thought to be indispensable for maturation and synaptogenesis (Kishi and Macklis, 2005; Guy et al., 2011).

Different types of breathing abnormalities are associated with defects of MeCP2. Female RTT patients show periods of hyperventilation alternating with prolonged periods of breath-holdings (Kerr and Julu, 1999; Julu et al., 2001). In contrast, male patients rather show hypoventilation, apneas and respiratory insufficiency soon after birth (Geerdink et al., 2002; Kankirawatana et al., 2006; Schüle et al., 2008). The male *Mecp2^−/y^* null mice, which are the original model to analyze the loss of MeCP2 in neurons, have a characteristic impairment of breathing (Guy et al., 2001), which manifests as hypoventilation, with a reduced respiratory rate and minute ventilation together with a high number of apneas (Viemari et al., 2005; Chao et al., 2010; Wegener et al., 2014). Currently, the ultimate cause of altered respiratory behavior is not yet known and might very well be different in male and female subjects. However, it is clear that breathing of MeCP2 deficient mice is influenced by many factors that include neurotransmitter systems like norepinephrine (Viemari et al., 2005) or neurotrophic factors like BDNF (Li and Pozzo-Miller, 2014) as well as cellular systems like glia cells (Lioy et al., 2011; Delépine et al., 2015) and inhibitory neurons (Hülsmann et al., 2016).

When we previously investigated the serotonergic system, we found that the serotonin receptor 5-ht_5b_ is heavily dysregulated in the brainstem of *Mecp2^−/y^* mice (Vogelgesang et al., 2017). Rodents have been shown to possess two functional 5-ht_5_ receptor subtypes, 5-ht_5a_ (Plassat et al., 1992) and 5-ht_5b_ (Matthes et al., 1993). While their physiological role is unknown, both are expressed at low levels in several brain regions and appear to be restricted to neural tissue (Rees et al., 1994). So far, we know that 5-ht_5b_ is expressed as both a full length as well as a truncated protein that is retained in the endosomal compartment. Yet, it is still able to interact with proteins (Vogelgesang et al., 2017), and therefore can potentially alter second messenger signaling and cAMP levels.

In wild type (WT) mice, the expression of 5-ht_5b_ receptor gradually increases during early development, peaks around postnatal day 21 (P21) and is then down-regulated. In *Mecp2^−/y^* mice, however, 5-ht_5b_ expression remains elevated past P21 (Vogelgesang et al., 2017). This developmental difference coincides with the appearance of the respiratory phenotype, which develops between P20 and P40 (Viemari et al., 2005; Mesuret et al., 2018).

To investigate the functional role of the persistent expression of 5-ht_5b_ receptors *in vivo* we generated MeCP2-deficient mice that also lack the 5-ht_5b_ receptor (*Mecp2^−/y^*;*Htr5b*^−/−^ mice). Since our goal was to investigate the role of 5-ht_5b_, we chose to limit our analysis to male *Mecp2^−/y^*;*Htr5b*^−/−^ *mice, because* the breathing phenotype of female mice is less predictable due to the variable x-chromosomal inactivation (Johnson et al., 2015). Moreover, female mice remain asymptomatic often for more than a year (Guy et al., 2001; Wegener et al., 2014). Using only male mice allowed us not only to analyze the effect of the 5-ht_5b_ receptors on breathing but also to measure their impact on the cellular cAMP level in the medulla.

## Materials and Methods

### Ethics Statement

The experimental procedures were performed in accordance with European Community (EU Directive 2010/63/EU for animal experiments) and National Institutes of Health guidelines for the care and use of laboratory animals. In accordance with the German Protection of Animals Act (TierSchG §4 Abs. 3) procedures were approved by the Animal Welfare Office of University Medical Center Gottingen (file number ID T12/18).

### Animal Models

The knockout mouse model for RTT, strain B6.129P2(C)-Mecp2tm1-1Bird (Guy et al., 2001; maintained on a C57BL/6J background) was purchased from The Jackson Laboratory (Bar Harbor, ME, USA). Hemizygous mutant *Mecp2^−/y^* males were generated by crossing heterozygous *Mecp2*^+/−^ females with C57BL/6J wild-type males. The genotyping was performed in accordance with The Jackson Laboratory genotyping protocols^1^.

The knockout mouse model for the 5-ht_5b_ receptor, strain 129SvEvBrd (maintained on a 29/SvEv-C57BL/6 background) was obtained from Taconic Europe A/S (Tornbjergvej 40, Ejby, 4623 Lille Skensved, Denmark). *Htr5b* knockout mice were backcrossed into the C57BL/6 background for at least eight generations. *Mecp2^−/y^*;*Htr5b*^−/−^-double-knockout mice were then generated by crossbreeding female *Mecp2*^+/−^;*Htr5b*^+/−^ mice with male *Mecp2^+/y^*;*Htr5b*^−/−^ mice.

### Genotyping

Tissue samples were incubated in 25 mM NaOH/0.2 mM EDTA for 3 h at 65°C. After neutralization with an equal volume of 40 mM Tris/HCl pH 5.5, 1 μL was taken as a template for subsequent PCR. The primers used for the identification of *Htr5b*-genotype were: WT-for (5′-ctctgcagtcggtttgatg-3′), WT-rev (5′-gtagagtcaccacaagcac-3′), KO-for (5′-gcagcgcatcgccttctatc-3′), KO-rev (5′-gtgctgggattagaagtcc-3′). The primers used for the identification of the *Mecp2* genotype were: WT-for (5′-gaccccttgggactgaagtt-3′), KO-for (5′-ccatgcgataagcttgatga-3′) and WT-KO-rev (5′-ccaccctccagtttggttta-3′).

### Unrestrained Whole-Body- Plethysmography

Ventilation was measured by unrestrained whole-body-plethysmography (Drorbaugh and Fenn, 1955; Bartlett and Tenney, 1970) in 40-day old mice (P40). Mice were placed in a custom-made acrylic glass chamber (300 ml) that was connected to a differential low-pressure transducer (model DP1 03, Validyne Engineering, Northridge, CA, USA). The second channel of the pressure transducer was connected to a reference chamber (300 ml). The signal from the pressure transducer was fed into a sine wave carrier demodulator (CD-15, Validyne Engineering). Animals could explore the chamber freely. For the analysis, pressure changes were band-pass filtered (1.5–500 Hz) and amplified (four times) before storing on an Apple-PC computer. For digitization (1 kHz sampling rate) an ITC-16 interface (InstruTECH/HEKA, Lambrecht) was used that was controlled by Axograph 4.8 software (Axon Instruments, Foster City, CA, USA). A bias flow of 150 ml/min was introduced using a Normocap^®^ CO_2_-sensor (Datex, Instrumentarium Oy, Helsinki, Finland). Pressure changes were exported and converted to axon binary files and used for analysis. Breaths (Inspiratory flow peaks) from a period of 3 min after 12 min adaptation to the chamber were analyzed by the threshold search peak detection method of Axon Clampfit (Molecular Devices, Sunnyvale, CA, USA). We did not discriminate between respiratory cycles associated to different types of behavior e.g., sniffing. Breathing frequencies were calculated as the reciprocal of the averaged inspiratory peak interval. The number of intervals between inspiratory peaks that were longer than 1 s was determined during the 3 min as a parameter for central apneas (Stettner et al., 2008). To define the regularity of breathing, the coefficient of variation was calculated for the interval (CV = STD/mean) as well as an irregularity score (IS) was calculated: IS = 100*ABS[(Int_n_ − Int_n-1_)/Int_n-1_] for each respiratory cycle (Barthe and Clarac, 1997; Telgkamp et al., 2002).

### Measurement of cAMP

The cAMP concentration of murine tissue was determined using commercially available ELISA-based DetectX-kit (Arbor Assays) and performed according to manufacturer’s instructions. Absorbance was measured at 450 nm using Infinite 200 Pro reader (TECAN). Whole brains were explanted from 40 days old mice, separated and individual regions snap-frozen in liquid nitrogen.

### Statistics

The cAMP measurements and plethysmography were analyzed with non-parametric Kruskal-Wallis and subsequent Dunn’s *post hoc* test for multiple comparisons. The survival plots were analyzed using the log-rank (Mantel-Cox) and Gehan-Breslow-Wilcoxon tests. Analyses were performed using GraphPad Prism version 5.0d for Mac OSX (GraphPad Software, San Diego, CA, USA). Differences were considered statistically significant at *P* < 0.05. Data are presented as the mean ± standard error of the mean (SEM; *n* = number of experiments).

## Results

### Effect of 5-ht_5b_ Knockout *in Vivo*

**Figure 1:**
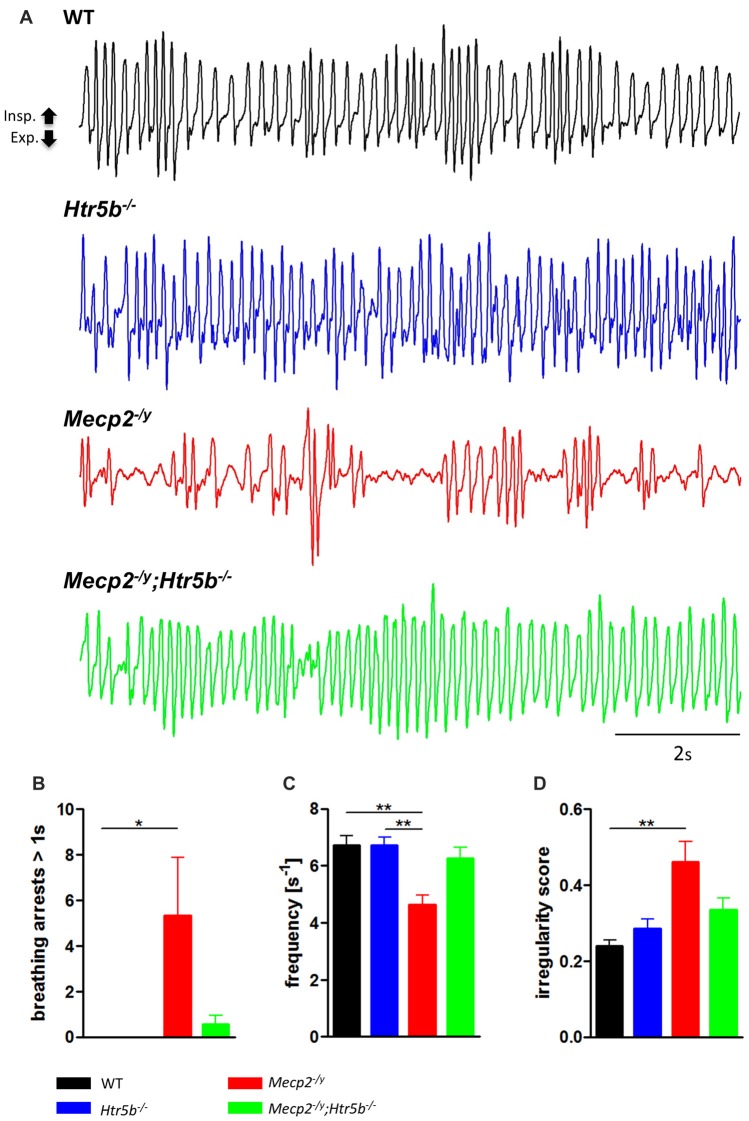
Plethysmographic *in vivo* recordings. **(A)** Plethysmographic *in vivo* recordings of wild type (WT; *n* = 9), *Htr5b*^−/−^ (*n* = 7), *Mecp2^−/y^* (*n* = 10) and *Mecp2^−/y^*;*Htr5b*^−/−^ (*n* = 11) mice at postnatal day 40 (P40). Recordings revealed improved breathing in *Mecp2^−/y^*;*Htr5b*^−/−^ mice, which was expressed by the **(B)** reduction of long (>1 s) apneas, **(C)** respiratory frequency and **(D)** irregularity score (IS). Corresponding bar diagrams represent the mean value and standard error of the mean (SEM). Asterisks indicate significance (*P < 0.05, **P < 0.01; Kruskal-Wallis test with Dunn’s multiple comparisons).

*Mecp2^−/y^*;*Htr5b^−/−^* mice were generated by crossbreeding female *Mecp2^+/−^*; *Htr5b*^+/−^ mice with male *Mecp^2+/y^*; *Htr5b*^−/−^ mice. To our knowledge, *Htr5b*^−/−^ mice have no observable phenotype (see www.taconic.com and Mouse Genome Database, http://www.informatics.jax.org/marker/MGI:96284). In our hands, the life span of *Htr5b*^−/−^ mice was normal and body weight at P40 (19.06 ± 1.12 g) was indistinguishable from WT mice (20.60 ± 0.56 g) of the same age. Unrestrained whole-body plethysmography at postnatal day 40 (P40) revealed that *Htr5b*^−/−^ mice were breathing normal, which is in line with the notion that 5-ht_5b_-receptors are downregulated and thus dispensable at this age (Figure 1A). Like WT mice (*n* = 9), *Htr5b*^−/−^ mice (*n* = 7) did not show long (longer than 1 s) breathing arrests (Figure 1B), which are typical for *Mecp2^−/y^*, and both, breathing rate (WT 6.68 s^−1^ ± 0.41 vs. *Htr5b^−/−^* 6.69 s^−1^ ± 0.36; n.s.; Figure 1C) and IS (WT 0.23 ± 0.03 vs. *Htr5b*^−/−^ 0.28 ± 0.02; n.s.; Figure 1D) were indistinguishable from WT.

### Breathing of Mecp2^−/y^;*Htr5b*^−/−^ Mice Lacks Respiratory Arrests

**Figure 2:**
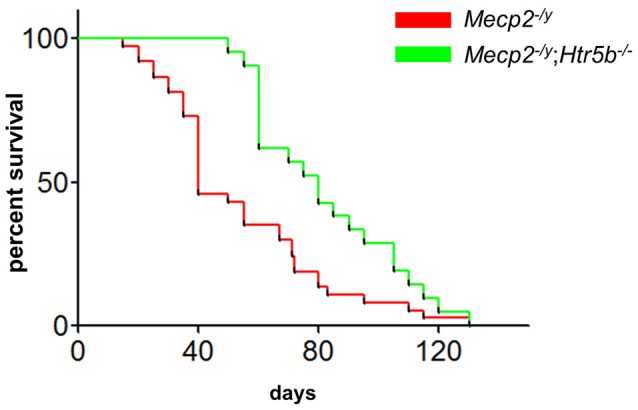
*Mecp2^−/y^*;*Htr5b^−/−^* mice show improved survival. The survival plot of *Mecp2^−/y^* (red; *n* = 37) vs. *Mecp2^−/y^*;*Htr5b*^−/−^ (green; *n* = 21) mice revealed a significantly increased median lifespan of 40–80 days, respectively (*p* = 0.0058 (Mantel-Cox) or *p* < 0.001 (Gehan-Breslow-Wilcoxon). WT and *Htr5b*^−/−^ mice showed no lethality in the timeframe indicated and were omitted for clarity.

When comparing breathing of *Mecp2^−/y^*;*Htr5b^−/−^* mice (*n* = 11) with *Mecp2^−/y^* mice (*n* = 10), we found significantly improved breathing parameters, yet no full rescue. Unrestrained whole-body plethysmography at P40 revealed a slow irregular breathing rate in *Mecp2^−/y^* mice with a high number of breathing arrests (5.3 ± 2.6 per 180 s, Figure 1B), low respiratory rate (4.6 ± 0.36 s^−1^; Figure 1C) and high IS (0.46 ± 0.06; Figure 1D). However, the breathing rhythm of *Mecp2^−/y^*;*Htr5b*^−/−^ mice was more stable with intermediate values between *Htr5b*^−/−^ (*or* WT) *and Mecp2^−/y^* mice. *Mecp2^−/y^*;*Htr5b*^−/−^ mice showed a significantly lower number of breathing arrests longer than 1 s (0.5 ± 0.4 per 180 s; Figure 1B), and a higher respiratory rate (6.23 ± 0.65 s^−1^; Figure 1C), and the IS (0.33 ± 0.03; Figure 1D) was indistinguishable from WT and *Htr5b^−/−^* mice. Moreover, body weight of *Mecp2^−/y^*;*Htr5b*^−/−^ mice (17.52 ± 0.75 g) was higher than age-matched *Mecp2^−/y^* mice (12.31 g ± 0.8; *p* < 0.001) but did not reach the level of WT and *Htr5b*^−/−^ mice. The life span of *Mecp2^−/y^*;*Htr5b*^−/−^ mice was improved compared to *Mecp2*^−/y^ mice (median 80 vs. 40 days), however no *Mecp2^−/y^*;*Htr5b*^−/−^ survived longer than 130 days (Figure 2).

### 5-ht_5b_ Affects cAMP Levels in Brainstem

**Figure 3:**
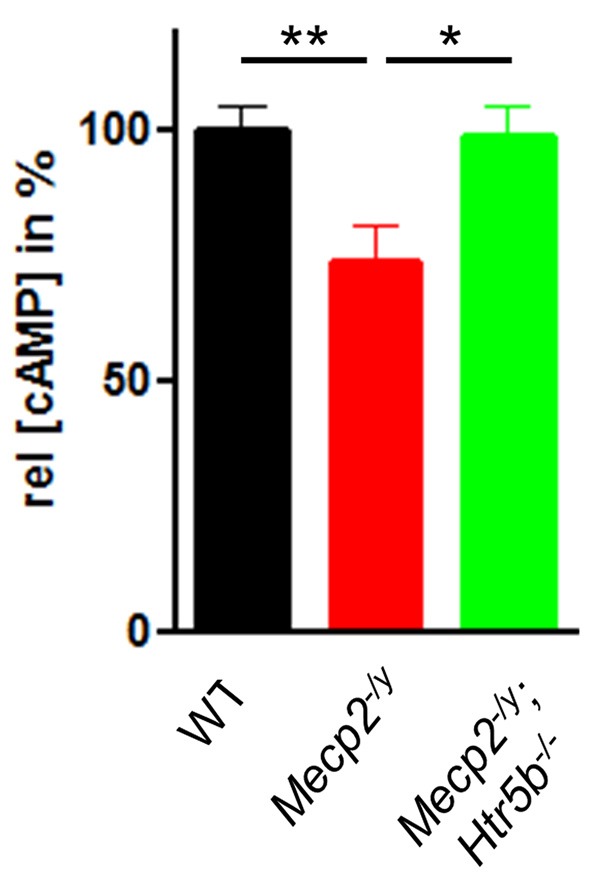
Effect of *Htr5b* expression on cAMP levels. cAMP concentration in brainstem tissue of WT (*n* = 5), *Mecp2^−/y^* (*n* = 5) and *Mecp2^−/y^*;*Htr5b*^−/−^ (*n* = 5) mice. The bar diagram illustrates the relative cAMP concentration ([cAMP]) in brainstem tissue at P40 of *Mecp2^−/y^* knockout mice and *Mecp2^−/y^*;*Htr5b*^−/−^ double knockout mice in comparison to WT mice at P40. Asterisks indicate significance (*P < 0.05, **P < 0.01; Kruskal-Wallis test with Dunn’s multiple comparisons).

Since 5-ht_5b_ receptors are highly expressed in the brainstem of MeCP2-deficient mice at P40 (Vogelgesang et al., 2017) and 5-ht_5b_ receptors are able to reduce cellular cAMP levels *in vitro* (Niebert et al., 2017), we measured the cAMP concentration in medullary brainstem lysates. The cAMP concentration of *Mecp2^−/y^* mice at P40 was significantly lower (73.49 ± 7.27%) when compared to WT mice (Figure 3), whose cAMP levels were set as 100%. This reduction is in line with previous reports of low cAMP in the brainstem of *Mecp2^−/y^* mice (Mironov et al., 2011). In agreement with the hypothesis of constitutive 5-ht_5b_-receptor signaling we found normal cellular cAMP in *Mecp2^−/y^*; *Htr5b*^−/−^ mice (98.46 ± 6.34%; Figure 3). These data are in line with the concept that the constitutive activity of 5-ht_5b_ is impairing cAMP signaling in *Mecp2*^−/y^ mice (Vogelgesang et al., 2017).

## Discussion

The *Mecp2^−/y^* mouse model of RTT shows a prominent respiratory phenotype that develops relatively late during postnatal development. At P40, breathing is more irregular and MeCP2-deficient mice show reduced respiratory rate and an increased number of longer breathing arrests (Janc et al., 2016). At this age, 5-ht_5b_ receptor expression in the medulla of *Mecp2*^−/y^ mice is still elevated while it drops to neonatal level in the WT littermates between P21 and P40 (Vogelgesang et al., 2017). In the present study, we tried to test for a pathophysiological link between the persistence of the 5-ht_5b_ receptor and the breathing disturbances using mouse genetics.

### Alteration of Neuromodulator Pathways

5-ht_5b_ receptors are not targeted to the plasma membrane but to the endosomal compartment, where they can participate in signaling by binding to G_i_ proteins (Vogelgesang et al., 2017) and lowering cAMP levels (Niebert et al., 2017). Lower cAMP-levels in Mecp2^−/y^ mice provide a simplistic rationale for the clinical use of cAMP-elevators like theophylline, as the “first choice for respiratory stimulation” in Rett patients (Julu et al., 2008). In addition, the resulting imbalance of cAMP-dependent second messenger cascades is likely to affect not only the serotonergic system (Manzke et al., 2003) but other neurotransmitters that have been implicated in the modulation of the respiratory network (Fujii et al., 2004; Lalley, 2008; Viemari, 2008; Mellios et al., 2014). In this respect, other effects on cAMP may seem contradictory: application of 5-HT_1A_ receptor agonists, which reduces cAMP, has been reported to have positive effects on respiration in a Rett mouse model (Abdala et al., 2010, 2014) and patients (Andaku et al., 2005; Ohno et al., 2016). However, interactions between different neuromodulator pathways are manifold and even two G_i_-mediated pathways can be antagonistic if restricted to different cellular compartments or located in distinct types of neurons (Manzke et al., 2010). Additionally, 5-HT_1A_ receptors can modulate neuronal activity independently from cAMP, e.g., by modulating potassium currents (Penington et al., 1993). Although we found a net reduction of cAMP in the medulla, we cannot rule out an additional effect of the 5-ht_5b_ dependent reduction of 5-HT_1A_ receptor surface expression (Niebert et al., 2017). However, this mechanism appears rather unlikely to be of major importance, since 5-HT_1A_ knockout mice have only a mild respiratory phenotype (Barrett et al., 2012). Moreover, 5-HT_1A_ receptor agonists have diverse effects including an anxiolytic action that, in the light of a potential role of the anxiety level of *Mecp2^−/y^* mice (Ren et al., 2012), might influence respiratory rhythm without directly influencing respiratory neurons.

### Survival Is Still Impaired

The fact that the improvement of breathing does not completely rescue the life expectancy of the mice is, although disappointing, in line with earlier observations, as for the restoration of MeCP2 in inhibitory neurons (Hülsmann et al., 2016). In *Mecp2^−/y^;*Htr5b*^−/−^* double-knockout mice, body weight was still reduced compared to WT and *Htr5b^−/−^* mice and, although increased, the lifespan of *Mecp2^−/y^*;*Htr5b*^−/−^ double-knockout mice was still significantly shorter than the lifespan of WT mice. Since we did not find a dysregulation of the 5-ht_5b_-receptor expression in the hippocampus (Vogelgesang et al., 2017), any hippocampal pathology, which is e.g., involved in the increased propensity to seizures (Boison, 2012), is not cured in *Mecp2^−/y^*;*Htr5b*^−/−^ mice and, thus, can manifest later during the development of *Mecp2^−/y^* mice leading to still premature death.

### Translation to Human Rett Syndrome

Although the persistent expression of 5-ht_5b_ contributes to the respiratory phenotype in MeCP2-deficient mice, these findings cannot be immediately translated to human patients. Unlike in mice (Grailhe et al., 2001; Maekawa et al., 2010; Vogelgesang et al., 2017), it is accepted that stop codons in the human *HTR5B* gene prevent its expression (Grailhe et al., 2001). However, we found that 5-ht_5b_ in mice is also expressed as a truncated protein (Vogelgesang et al., 2017). As nothing is known about the expression and potential splice variants of 5-ht_5b_ in humans, further analysis is required to identify potential alterations of 5-ht_5b_ receptor expression in the brainstem of patients with MECP2 mutations.

### Summary

MeCP2 deficiency affects several 100 targets (Ben-Shachar et al., 2009) so any effect of 5-ht_5b_-receptor and cAMP signaling must be seen in the context of multiple other dysregulated genes. Our data supports the notion that 5-ht_5b_-receptor dysregulation is an important but probably not the only factor that contributes to respiratory problems in *Mecp2^−/y^* mice. However, currently no data is available indicating a relevance of the 5-ht_5b_ receptor expression in the pathology of human RTT patients.

## Author Contributions

SV, AMB, TM and SH performed the experiments. TM, AMB, MN and SH analyzed the data. MN and SH wrote the manuscript.

## Conflict of Interest Statement

The authors declare that the research was conducted in the absence of any commercial or financial relationships that could be construed as a potential conflict of interest.

## Footnotes

^1^http://jaxmice.jax.org/strain/003890.html
